# Diagnostic Accuracy, Effectiveness and Cost for Cognitive Impairment and Dementia Screening of Three Short Cognitive Tests Applicable to Illiterates

**DOI:** 10.1371/journal.pone.0027069

**Published:** 2011-11-02

**Authors:** Cristóbal Carnero-Pardo, Beatriz Espejo-Martínez, Samuel López-Alcalde, María Espinosa-García, Carmen Sáez-Zea, Elisa Hernández-Torres, José Luis Navarro-Espigares, Rosa Vílchez-Carrillo

**Affiliations:** 1 Servicio de Neurología, Hospital Universitario Virgen de las Nieves, Granada, Spain; 2 FIDYAN Neurocenter, Granada, Spain; 3 Servicio de Neurología, Complejo Hospitalario La Mancha Centro, Alcázar de San Juan, Spain; 4 Departamento de Psicología, Universidad de Jaén, Jaén, Spain; 5 Subdirección de Control de Gestión, Hospital Universitario Virgen de las Nieves, Granada, Spain; University Hospital La Paz, Spain

## Abstract

**Background:**

Illiteracy, a universal problem, limits the utilization of the most widely used short cognitive tests. Our objective was to assess and compare the effectiveness and cost for cognitive impairment (CI) and dementia (DEM) screening of three short cognitive tests applicable to illiterates.

**Methods:**

Phase III diagnostic test evaluation study was performed during one year in four Primary Care centers, prospectively including individuals with suspicion of CI or DEM. All underwent the Eurotest, Memory Alteration Test (M@T), and Phototest, applied in a balanced manner. Clinical, functional, and cognitive studies were independently performed in a blinded fashion in a Cognitive Behavioral Neurology Unit, and the gold standard diagnosis was established by consensus of expert neurologists on the basis of these results. Effectiveness of tests was assessed as the proportion of correct diagnoses (diagnostic accuracy [DA]) and the kappa index of concordance (k) with respect to gold standard diagnoses. Costs were based on public prices at the time and hospital accounts.

**Results:**

The study included 139 individuals: 47 with DEM, 36 with CI, and 56 without CI. No significant differences in effectiveness were found among the tests. For DEM screening: Eurotest (k = 0.71 [0.59–0.83], DA = 0.87 [0.80–0.92]), M@T (k = 0.72 [0.60–0.84], DA = 0.87 [0.80–0.92]), Phototest (k = 0.70 [0.57–0.82], DA = 0.86 [0.79–0.91]). For CI screening: Eurotest (k = 0.67 [0.55–0.79]; DA = 0.83 [0.76–0.89]), M@T (k = 0.52 [0.37–0.67]; DA = 0.80 [0.72–0.86]), Phototest (k = 0.59 [0.46–0.72]; DA = 0.79 [0.71–0.86]). There were no differences in the cost of DEM screening, but the cost of CI screening was significantly higher with M@T (330.7±177.1€, mean±sd) than with Eurotest (294.1±195.0€) or Phototest (296.0±196.5€). Application time was shorter with Phototest (2.8±0.8 min) than with Eurotest (7.1±1.8 min) or M@T (6.8±2.2 min).

**Conclusions:**

Eurotest, M@T, and Phototest are equally effective. Eurotest and Phototest are both less expensive options but Phototest is the most efficient, requiring the shortest application time.

## Introduction

Short cognitive tests (SCTs) are routinely applied by healthcare professionals in different settings. SCTs are usually associated with screening for cognitive impairment (CI) and dementia (DEM) and are also used to assess the response to treatment and to follow up this type of patient. Their results sometimes serve as criteria for access to studies, treatments, and disability benefits, among others.

Some of the most widely used SCTs, such as the *Memory Impairment Screen* [1] and *Addenbrooke's Cognitive Examination* [2], include tasks that require reading and writing ability and cannot be used on illiterates. Other tests, including the *Clock-Drawing Test* [3], *General Practitioner Assessment of Cognition* [4], *Mini-Cog* [5], *Montreal Cognitive Assessment* [6], *Seven Minute Test* [7], and *Rowland Universal Dementia Assessment Scale* [8], do not require reading or writing but involve the use of pencil and paper, which can generate aversion among illiterates and individuals of low educational level. The most frequently applied test, the *Mini-Mental State Examination* (MMSE) [9], has both types of item, making it especially unsuitable. In fact, its low validity and diagnostic accuracy in populations with a low educational level has been demonstrated by our group [10] and other authors [11], [12]. Educational factors are known to exert an important influence on MMSE results [13], [14], leading some authors to advise against its use in less-educated populations [15], [16].

In 2010, approximately 750 million individuals worldwide were estimated to be illiterate[17], in developed as well as developing nations. In the USA, 3% of adults (7 million) are illiterate [18]. Account must also be taken of the emergence of “relative illiteracy”, i.e., the inability of people literate in their own languages to read or write in the language of their host country. This phenomenon is on the increase, due to rises in emigration and tourism. Instruments requiring the ability to read cannot be used on people in this situation, considered to represent 2% of the USA population, around four million individuals [18].

Furthermore, CI and DEM are increasing in developing countries [19], where illiteracy is more widespread, underscoring the need for tests that do not need reading or writing skills or the use of pencil and paper, allowing whole populations to be evaluated with the same instruments.

The *Short Portable Mental Status Questionnaire* [20] and *Abbreviated Mental Test* [21] can be applied to an individual who is not literate and do not require pencil and paper, but they are of limited usefulness to detect CI. *The Leganes Cognitive Test* [22] and the *Community Screening Interview for Dementia* (CSI-D) [23], whose use was proposed by the 10/66 group [24], also have these illiterate-friendly characteristics, although they take too long to apply (>10 min) given the time constraints on Primary Care (PC) consultations [25].

Three new SCTs with none of these limitations are increasingly used in our country (Spain), in which more than 10% of the population aged over 65 years is illiterate due to particular historical circumstances [26]. One is the Eurotest [27] (www.eurotest.es), which evaluates knowledge and use of the local currency and includes an episodic memory test. Its results are not influenced by educational level, and it has proven to be a reliable and ecologically valid instrument [28]; it takes around 7 minutes to apply and yields information on the functional capacity and autonomy of the testee. Its discriminant validity for CI and DEM has been confirmed in various studies, including a meta-analysis [29], [30], [31], [32]. It can be used without any modification in all countries of the Eurozone and can be readily adapted to other official currencies [33].

Another of these recent SCTs, the Memory Alteration Test (M@T) [34], was designed for the early detection of Alzheimer's Disease, including prodromal stages [35], and it evaluates temporal orientation and different types of memory (episodic, textual, and semantic). It takes 5–6 minutes to apply and has a high internal consistency and validity, although no data are available on its reliability. Its results are mildly influenced by educational level, and distinct cutoff points according to years of schooling are recommended when used for CI screening (36/37 if <8 years; 37/38 if >8 years).

Finally, the Phototest [36] (www.fototest.es) is a very simple and short instrument (<3 min) that assesses naming, verbal fluency (people's names [37]), and episodic memory. Its results are normally distributed and are not influenced by educational level [38]. It has good test-retest and inter-observer reliability [38], and various studies have demonstrated that the cut-off points 26/27 and 28/29 offer adequate discriminant validity for DEM and CI, respectively [10], [31], [36], [39].

These three instruments differ widely in structure and application requirements but are all short, applicable to illiterates, performed without paper or pencil, and validated for CI (**Table 1**). The objective of this study was to compare the diagnostic accuracy, effectiveness and costs of DEM and CI screening with the use of these tests.

**Table 1:** Characteristics of the short cognitive tests.

		Structure					Instrumentation		Cut-off point	
Test	T	O	M	F	N	C	RS	Other	DEM	CI
**Eurotest**	5–10		X			X	X	Coins	20/21	22/23
**M@T**	5–10	X	X				X		28/29	36/37&37/38*
**Phototest**	<5		X	X	X		X	Laminated sheet	26/27	28/29

M@ T: Memory Alteration Test. T: time in minutes. O: orientation; M: memory; F: verbal fluency; N: naming; C: calculation. RS: record sheet. DEM: dementia; CI: cognitive impairment. *36/37 for individuals without and 37/38 for those with primary schooling.

## Methods

### Ethics Statement

The study was approved by the Ethics and Research Committee of the Virgen de las Nieves University Hospital (Granada, Spain), and written informed consent was obtained from all participants or their carers.

### Design

This prospective and naturalistic Phase III study of diagnostic test assessment [40] had a paired design (all tests applied to all participants) and complete verification (all participants underwent the standard diagnostic procedure) [41].

### Setting

Four PC centers in the Metropolitan District of North Granada Area (Southern Spain).

### Study population

Consecutive patients attended in PC centers from February 1 2008 to January 31 2009 with suspicion of CI or DEM based on observations of healthcare professionals or reports of memory or cognitive impairment problems by patient, family member, or a third party. Exclusion criteria were absence or withdrawal of consent, previous enrolment in this study, or previous diagnosis of CI or DEM. There were no age limits. Sensory or motor deficits or other prior conditions were not reasons for exclusion.

### Procedure

The Eurotest, M@T, and Phototest were applied in a balanced manner to all participants. Regardless of their test results, all subsequently visited the Cognitive Behavioral Neurology Unit (CBNU) of the Neurology Department of Virgen de las Nieves University Hospital, Granada for a complete assessment, including clinical examination and behavioral testing (Spanish adaptation of the *Neuropsychiatric Inventory Questionnaire* [42]), functional testing (*Barthel Index* [43], *Lawton-Brody* scale [44] and *Functional Activities Questionnaire* [45]), and a detailed neuropsychological examination on orientation, attention (direct and inverted series), executive function (similarities, verbal fluency), memory (learning, deferred recall and recognition), language (naming, comprehension, fluency), visual-spatial functions [drawing, copying], calculation, and motor praxis. Two neurologists with experience in cognitive-behavioral neurology established the gold standard diagnosis by consensus, based on the CBNU evaluations, classifying individuals as: Non-CI (NoCI), CI non-DEM (CInD) (criteria for mild CI of the Neurology of Behavior and Dementias Study Group of the Spanish Neurology Society [46]) or DEM (DSM-IVR criteria) [47]. Any lack of consensus was resolved by the decision of a third neurologist. All three experts were blinded to the screening test results.

### Statistical analysis

The effectiveness of the screening tests was evaluated by analyzing the diagnostic accuracy (DA, proportion of correct diagnoses) and the kappa index of diagnostic concordance (k) with respect to the gold standard diagnosis (positive/negative), considering the cutoff points recommended by the authors (Table 1) [48]. Effectiveness was calculated for DEM *versus* non-DEM (NoCI+CInD) and for CI (DEM+CInD) *versus* NoCI.

The cost analysis was based on the perspective of our healthcare organization and took no account of the direct costs of patients/carers, indirect or intangible costs, or the economic effects of diagnostic delay due to false negatives. For each test, we considered the minimum costs required to reach the correct diagnosis for each condition. Hence, in the usual clinical situation, true negatives would only incur the cost of the PC consultation (43.5€), whereas true positives and false positives would also incur the costs of the CBNU study (neurologist, neuropsychologist, nurse: 197.4€) and of the minimum complementary tests recommended by the Spanish Neurology Society (analytical, cranial CT-scan: 193.4€), a total of 390.8€. For their part, false negatives would require at least one more PC consultation besides the initial evaluation, as well as the CBNU study. The cost of the PC consultation was taken from published rates and prices of the Andalusian public healthcare system (Order of October 14 2005), and the cost of the CBNU evaluation was based on the hospital's financial accounts (**Table 2**).

**Table 2:** Minimum costs per diagnosis.

	PC study	CBNU study	Total
Diagnoses	43.5€	390.8€	(€)
True Negative	1	-	43.5
True Positive	1	1	434.4
False Positive	1	1	434.4
False Negative	2	1	477.9

PC: Primary Care; CBNU: Cognitive Behavioral Neurology Unit.

SPSS version 15.0 (SPSS Inc., Chicago, IL) was used for the data analyses. Qualitative variables were compared among groups by means of the chi-square test or comparison of proportions, and quantitative variables were compared with an ANOVA, applying the Bonferroni test in *post-hoc* analyses. Time intervals and costs were compared with a t-test for related samples. P<0.05 was considered significant, and 95% confidence intervals were calculated for all study variables.

### Formal aspects

The study design and report writing complied with STARD recommendations for diagnostic test studies [41] and the Food and Drug Administration guidelines for reporting diagnostic study results [49].

## Results

**Figure 1:**
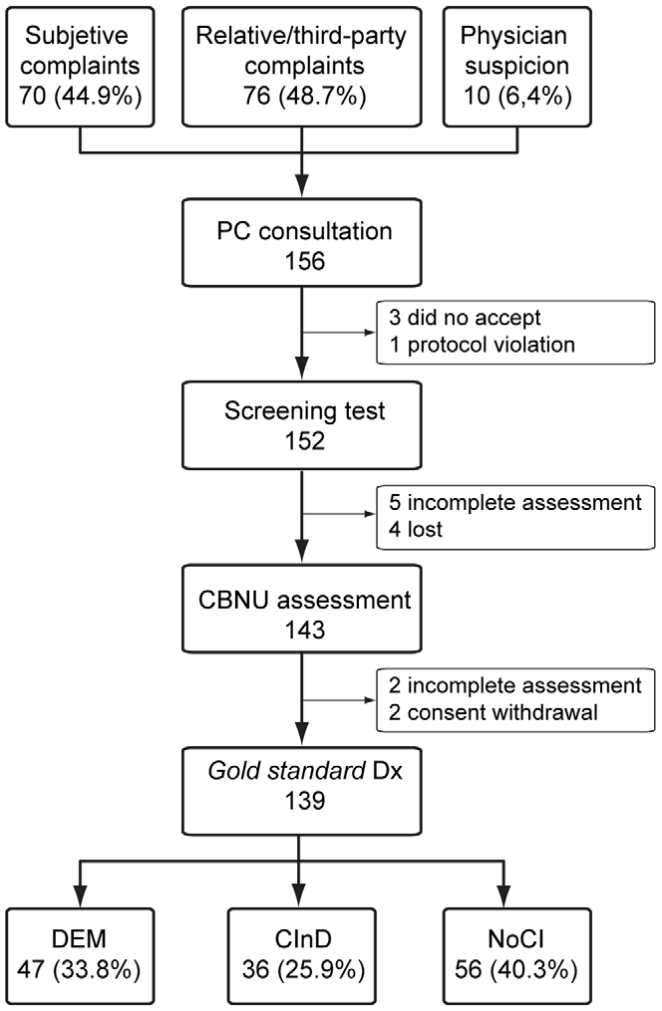
Study flow chart. PC: Primary Care; CBNU: Cognitive Behavioral Neurology Unit; DEM: dementia. CInD: cognitive impairment without dementia; NoCI: no cognitive impairment.

The four health centers in the study serve a population of 66,713 people, of whom around 16.7% are ≥65 years old [26]. During the study period, PC physicians reported suspicion of CI or DEM in 156 patients, based on complaints by the patients themselves in 70 cases (44.9%) and by a relative or third party in 76 cases (48.7%) or on initial observations by the physicians in the remaining 10 cases (6.4%). Out of these 156 patients, 17 did not complete the study: 5 refused or withdrew consent, 4 were lost to the follow up, 1 was excluded for protocol violation, and 7 were not fully evaluated, due to sequelae of stroke or traumatic brain injury associated or not with sensory deficit (amaurosis, cophosis) or recording error. Out of the 139 patients completing the study, 47 (33.8%) had DEM, 36 (25.9%) CI without DEM (CInD), and 56 (40.3%) had neither CI nor DEM (NoCI) (**figure 1**).

**Table 3** shows the socio-demographic characteristics and screening test results of the participants, stratified by cognitive diagnosis. The mean age (±sd) was 72.0±11.5 yrs; 72.7% were female. More than half (50.4%) had not completed primary schooling, and 14.4% of these were not literate. There were no significant gender differences among diagnostic groups, but subjects with DEM were older and had a lower educational level than those with CInD or NoCI (p<0.001 for all comparisons). The groups significantly differed in test results in the order NoCI>CInD>DEM (p<0.001 for all comparisons). The time taken to complete the test did not significantly differ (p = 0.06) between Eurotest (7.1±1.8 min) and M@T (6.8±2.2 min) but was significantly lower (p<0.001) for Phototest (2.8±0.8 min) than for either.

**Table 3:** Socio-demographic characteristics and screening test results by diagnostic group.

		Total	CI	NoCI	CInD	DEM	NoDEM
		(n = 139)	(n = 83)	(n = 56)	(n = 36)	(n = 47)	(n = 92)
**Age**	years	72.0±11.5	77.1±7.5	64.6±12.2	74.3±7.3	79.3±7.0	68.4±11.6
**Sex**	female	101 (72.7)	59 (71.1)	42 (75.0)	22 (61.1)	37 (78.7)	64 (69.6)
	male	28 (28.3)	24 (29.9)	15 (25.0)	14 (38.9)	10 (21.3)	28 (30.3)
**Educational level**	Illiterate	20 (14.4)	19 (22.9)	1 (1.8)	3 (8.3)	16 (34.0)	4 (4.3)
	<Primary	50 (36.0)	33 (39.8)	17 (30.4)	15 (41.7)	18 (38.3)	32 (34.8)
	≥Primary	69 (49.6)	31 (37.3)	38 (67.9)	18 (50.0)	13 (27.7)	56 (60.9)
**Eurotest**	Score	20.4±10.2	14.9±9.2	28.5±4.5	22.6±6.4	9.0±6.3	26.2±6.0
	time (min)	7.1±1.8	7.7±1.5	6.3±1.9	7.7±1.7	7.8±1.3	6.8±2.0
**Phototest**	Score	29.0±7.7	25.3±6.9	34.5±5.0	30.4±4.8	21.4±5.5	32.9±5.4
	time (min)	2.8±0.8	2.9±0.8	2.5±0.7	2.5±0.5	3.2±0.9	2.5±0.6
**M@T**	Score	29.0±12.5	22.2±11.0	39.2±6.0	31.2±8.3	15.3±7.3	36.1±8.0
	time (min)	6.8±2.2	7.6±2.0	5.6±1.9	6.6±1.5	8.4±1.9	6.0±1.8

CI: cognitive impairment (CInD+DEM). NoCI: no cognitive impairment. CInD: cognitive impairment without dementia. DEM: dementia. NoDEM: no dementia (NoCI+CInD). M@T: Memory Alteration Test. Data are n° individuals (percentage) or mean±sd.

All three tests showed very high and similar DA values for DEM (**table 4**): 0.86 (95% confidence interval [95%CI], 0.79–0.91) for Phototest and Eurotest and 0.87 (95%CI, 0.80 – 0.92) for M@T, with no significant difference among them (p = 0.94). Diagnostic concordance values were also substantial [50] and practically identical for DEM: 0.70 (95%CI, 0.57 – 0.82) for Phototest, 0.71 (95%CI, 0.59 – 0.83) for Eurotest, and 0.72 (95%CI, 0.60 – 0.84) for M@T (p = 0.97). For DEM, the instruments did not significantly differ in mean associated costs: 206.6±196.6€ for Phototest, 221.3±196.0€ for M@T, and 221.6±196.4€ for Eurotest (p>0.05, all comparisons).

**Table 4:** Effectiveness and cost for dementia.

Test	CuP	TP	TN	FP	FN	DA	k	Mean cost (€)
**Eurotest**	20/21	44	76	16	3	0.86 (0.79–0.91)	0.71 (0.59–0.83)	221.6±196.4
**M@T**	28/29	45	76	16	2	0.87 (0.80–0.92)	0.72 (0.60–0.84)	221.3±196.0
**Phototest**	26/27	38	82	10	9	0.86 (0.79–0.91)	0.70 (0.57–0.82)	206.6±196.6

M@T: Memory Alteration Test. CuP: cutoff point; TP: true positives; TN: true negatives; FP: false positives; FN: false negatives; DA: diagnostic accuracy (proportion of correct diagnoses); k: kappa índex. In parentheses: 95% confidence interval. Mean cost: mean±sd.

All three tests had a lower predictive value for CI than for DEM (**table 5**). For CI, no significant differences were found among the tests, with slightly but not significantly superior results for Eurotest (k = 0.67 [95%CI, 0.55 – 0.79]; DA = 0.83 [95%CI, 0.76 – 0.89]) *versus* M@T (k = 0.55 [95%CI, 0.43 – 0.71]; DA = 0.73 [95%CI, 0.64 – 0.80]) and Phototest (k = 0.59 [95%CI, 0.46 – 0.72]; DA = 0.79 [95%CI, 0.71 – 0.86]). For CI, mean associated costs did not significantly differ between Eurotest and Phototest (294.1±195.0€ vs. 296.0±196.5€; p = 0.79) but were significantly higher with M@T (330.7±177.1€) than with Eurotest (p<0.001) or Phototest (p<0.01).

**Table 5:** Effectiveness and cost for cognitive impairment.

Test	CuP	TP	TN	FP	FN	DA	k	Mean cost (€)
**Eurotest**	22/23	64	52	4	19	0.83 (0.76–0.89)	0.67 (0.55–0.79)	294.1±195.0
**M@T**	*	73	38	18	10	0.73 (0.67–0.80)	0.55 (0.43–0.71)	330.7±177.1
**Phototest**	28/29	58	52	4	25	0.79 (0.71–0.86)	0.59 (0.46–0.72)	296.0±196.5

M@T: Memory Alteration Test. CuP: cutoff point; TP: true positives; TN: true negatives; FP: false positives; FN: false negatives; DA: diagnostic accuracy (proportion of correct diagnoses); k: kappa índex. In parentheses: 95% confidence interval. Mean cost: mean±sd. *36/37 for individuals without primary schooling, 37/38 for those with at least primary schooling.

## Discussion

In this study of diagnostic test assessment in an urban population with a low educational level, all three SCTs evaluated showed a similar and substantial effectiveness [50], correctly classifying around 85% of the sample for DEM and 80% for CI. The instruments did not differ in mean associated costs in relation to DEM detection, while the cost of M@T for CI was slightly higher than that of Eurotest and Phototest, probably attributable to its lesser specificity, i.e., larger number of false positives. An important finding was that the Phototest is completed in half the time (<3 min) required by the other two tests (≈7 min), a significant and clinically relevant difference.

Our results are highly similar to those of individual studies of these instruments. In a meta-analysis, Eurotest [32] was found to correctly classify 85% of patients for DEM, virtually identical to the percentage (87%) in the present study and using the same cut-off value (20/21). The Phototest results for each diagnosis (34.5±5.0 NoCI, 30.4±4.8 CInD, 21.4±5.5 DEM) did not significantly differ from results obtained with the Phototest in a multi-center study using the same diagnostic criteria (33.1±4.8, 28,9±4.8 and 19,9±6.8, respectively) [39]. The mean M@T score of the present CInD group (31.2±8.3) is virtually identical to that of the group with mild CI (the only diagnosis with a comparable definition) in the original M@T study (31.5±3.9), in which 81% were correctly classified for CI, very close to the percentage obtained in the present study (78%) [34].

A recent secondary analysis of studies by the 10/66 Dementia Research Group reported that a shortened version of the CSI-D takes around five minutes to apply and offers the same diagnostic performance as the complete version of this instrument [51]. Youden index (sensitivity-[1-specificity]) scores for DEM ranged from 0.63 to 0.75 in all study groups except for one in Nigeria (0.92) [51]; these results, which have not yet been verified in a prospective study in the clinical setting, are very similar to the those obtained with each test in the present study (Eurotest 0.77, M@T 0.79, Fototest 0.70).

To our best knowledge, this is the first study of SCTs that evaluates the associated costs. Although not strictly a cost-effectiveness analysis [52], our report on the differences among these tests can serve as a guide for decision-making in the interests of maximizing efficiency. We did not take account of the material costs of applying the tests, on the assumption that they use an equal and minimal amount of resources, requiring just one sheet of paper plus a laminated sheet for Phototest and a set of coins for Eurotest (**Table 1**), which can both be reutilized. It should also be borne in mind that we adopted the perspective of our public health system, which is free to users. With regard to the key resource of time, all three are reasonably short, but Phototest needs only 3 minutes, less than half the time taken to apply Eurotest or M@T and highly suited to the usual time restrictions for a PC consultancy [25].

Phototest meets all recommended conditions to be utilized in PC [53], including: the favorable cost-effectiveness results found in this prospective study in the PC setting, the ease of its application [36], brevity (<3 min), and superior diagnostic usefulness in comparison to MMSE [10].

The main strength of this diagnostic test evaluation is its naturalistic and pragmatic nature, given that it was prospectively performed in the care setting in which the diagnostic problem arises, with a sufficiently long study period to avoid any seasonal variations. Moreover, the recruitment was consecutive and systematic, with minimal exclusion criteria. Consequently, our sample offers a faithful reflection of the diagnostic problem routinely faced in this care setting, yielding robust results that estimate the effectiveness rather than just the efficacy of these instruments. In addition, the main biases found in diagnostic test evaluations [40], [54] were minimized by evaluating all participants with all three tests and subjecting all to the gold standard diagnostic procedure, regardless of their screening test results.

One study limitation was the lack of a comparative analysis among diagnostic subgroups. A further potential weakness was the serial application of the tests, which may have produced biases due to fatigue, despite their short nature. To address this possibility, we applied the tests in a balanced manner, finding that the results were not affected by the order in which a test was taken (data not shown).

In conclusion, our results demonstrate that Eurotest, M@T and Phototest are equally effective instruments for DEM and CI screening in the PC setting. The cost of each test was similar for DEM identification, while the cost of M@T was slightly higher for CI screening. Phototest was applied in half the time required by the other two tests, a statistically significant and clinically relevant finding, establishing Phototest as the most efficient option and especially suitable for utilization in PC.
